# Evaluation of In Vivo and In Vitro Toxicity of Chestnut (*Castanea mollissima* Blume) Plant: Developmental Toxicity in Zebrafish Embryos Cytotoxicity, Antioxidant Activity, and Phytochemical Composition by LC‐ESI‐MS/MS

**DOI:** 10.1002/fsn3.70387

**Published:** 2025-06-11

**Authors:** İbrahim Demirtas, Mehmet Nuri Atalar, Zeynebe Bingol, Mine Köktürk, Gunes Ozhan, Amine Hafis Abdelsalam, Sevki Arslan, İlhami Gülçin

**Affiliations:** ^1^ Biochemistry Department, Faculty of Arts and Sciences Iğdır University Iğdır Türkiye; ^2^ Department of Pharmaceutical Chemistry, Faculty of Pharmacy Ondokuz Mayis University Samsun Türkiye; ^3^ Department of Nutrition and Dietetics, Faculty of Health Sciences Iğdır University Iğdır Türkiye; ^4^ Department of Medical Services and Techniques, Tokat Vocational School of Health Services Gaziosmanpasa University Tokat Türkiye; ^5^ Department of Organic Agriculture Management, Faculty of Applied Sciences Igdir University Igdir Türkiye; ^6^ Izmir Biomedicine and Genome Center Dokuz Eylul University Health Campus Izmir Türkiye; ^7^ Department of Molecular Biology and Genetics Izmir Institute of Technology Izmir Türkiye; ^8^ Biology Department, Faculty of Science Pamukkale University Denizli Türkiye; ^9^ Department of Chemistry, Faculty of Sciences Ataturk University Erzurum Türkiye; ^10^ Agri İbrahim Cecen University Rectorate Agri Türkiye

**Keywords:** antioxidant activity, *Castanea mollissima*, chestnut, cytotoxicity, LC‐ESI‐MS/MS, polyphenol

## Abstract

The search for novel therapeutic agents has led to increasing interest in natural products, driven by the recognition that they may offer safer and more sustainable alternatives to synthetic drugs. This study aims to fill the gap in knowledge regarding the biological activity and safety of the water extract of chestnut ( *Castanea mollissima* ) (chestnut), a plant species with a long history of use in traditional medicine, by conducting a comprehensive evaluation of its antioxidant, antidiabetic, and neuroprotective properties. This study presents a comprehensive analysis of the water extract of chestnut for the first time using various bioanalytical antioxidant methods. The extract's inhibitory effects on key enzymes like acetylcholinesterase (AChE), butyrylcholinesterase (BChE), and α‐glycosidase were evaluated due to their relevance in metabolic and neurodegenerative disorders such as diabetes and Alzheimer's disease. Developmental toxicity and cytotoxicity were assessed using zebrafish ( *Danio rerio* ) embryos to evaluate the extract's biological safety. The major phenolic compounds present in the extract were identified by liquid chromatography‐electrospray ionization tandem mass spectrometry (LC‐ESI‐MS/MS), revealing catechin, gallic acid, taxifolin, and epicatechin as the predominant constituents. Antioxidant capacity was determined through radical scavenging assays using 2,2‐diphenyl‐1‐picrylhydrazyl (DPPH^•^) and 2,2′‐azino‐bis(3‐ethylbenzothiazoline‐6‐sulfonic acid) (ABTS^•+^), alongside ferric (Fe^3+^), cupric (Cu^2+^), and Fe^3+^‐TPTZ (ferric‐tripyridyltriazine) reducing power assays. The findings highlight the significant antioxidant, antidiabetic, and neuroprotective potential of the chestnut water extract, supporting its prospective use in pharmaceutical and nutraceutical applications.

## Graphical abstract

This study presents a comprehensive analysis of the water extract of chestnut ( *Castanea mollissima* ) for the first time using various antioxidant methods. The inhibitory effects of the extract towards AChE, BChE, and α‐glycosidase were evaluated. Developmental toxicity and cytotoxicity were assessed using zebrafish ( *Danio rerio* ) embryos for the extract's biological safety. The major phenolic compounds present in the extract were identified by LC‐ESI‐MS/MS. Also, antioxidant capacity was determined by DPPH^•^ and ABTS^•+^ scavenging, Fe^3+^, Cu^2+^, and Fe^3+^‐TPTZ reducing assays.

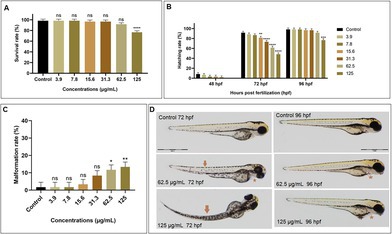

## 1 Introduction

Chestnut ( *Castanea mollissima* ) fruits are a pivotal food in many European countries and Turkiye (De Vasconcelos et al. 2010). Chestnut, widely distributed worldwide and also called the “king of dried fruits” (Zhang et al. 2014), is a monoecious plant with more male and fewer female flowers, with a distribution ratio of approximately 350:1. Taking advantage of this feature, nearly 90%–95% of the male flowers are removed to increase chestnut yield. With the recent progress of chestnut cultivation and chestnut product processing industry, the chestnut flowers yield has also increased in parallel. However, most of the chestnut flowers removed are not used due to insufficient research. Accordingly, a large resource is wasted. It is also known that chestnut flowers have been used effectively against cough, cold and diarrhea for a long time (Lim 2012; Carocho et al. 2014).

Plants and their fruits are the vital and native antioxidants sources that help improve and develop the properties of manufactured products in the food, cosmetics, and pharmaceutical sectors, and are also used for prevention of diseases in human metabolism. Natural antioxidants are of greater interest to consumers today because they have fewer side effects and are safer (Kalin et al. 2015; Adugna et al. 2024). Aromatic and medicinal plants and their fruits and flowers, which are sources of natural antioxidants, are also sources of phytochemicals with strong biological activities and many beneficial effects due to their strong antioxidant effects (Kızıltaş et al. 2021). Recent scientific and reliable studies have reported that natural antioxidants eliminate free radicals and prohibit the formation of reactive oxygen species (ROS) (Cetinkaya et al. 2012; Zhang et al. 2024). ROS are formed during incomplete reduction of oxygen and oxidative stress, and endogenous antioxidant compounds in the body and exogenous antioxidants taken from the diet balance their formation and keep their excessive production under control (Kandemir et al. 2017; Topal and Gulcin 2022). An imbalance between the antioxidant level in metabolism and ROS enhances the oxidative stress risk and, accordingly, ROS formation. In such a case, oxidative stress increases and different diseases begin to emerge (Gülçin et al. 2005; Karagecili et al. 2023). In this context, preventing ROS formation and suppressing oxidative stress are of vital importance in reducing inflammation and the formation of chronic diseases (Ekinci Akdemir et al. 2017; Bingol et al. 2021). Instead of widely used synthetic antioxidants including buthylated hydroxytoluene (BHT), *tert*‐butylhydroquinone(*t*‐BHQ) and buthylated hydroxyanisole (BHA), there is great interest and need for naturally sourced antioxidants to protect food products against oxidative stress and the metabolism against harmful ROS effects. After the scientific link between BHA and BHT and liver damage and cancer development was discovered, interest in synthetic antioxidants gradually decreased and serious restrictions were imposed on their use (Balaydin et al. 2010; Gülçin 2025). Therefore, there has been a tremendous demand for natural antioxidants, especially those from food sources that we get from outside in our diet.

Recent studies have demonstrated the existence of significant levels of bioactive compounds and antioxidants in diverse parts of the chestnut plant associated with antidiabetic activity, anticancer activity, cardiovascular disease, antioxidation and anti‐inflammatory abilities, and prevention of neurological dysfunctions (Baehaki et al. 2021; Akinyede et al. 2022; Peng, Yin et al. 2022). Especially since various extracts of chestnut flowers play a role in inhibiting lipid peroxidation and microbial growth, they are used in foods (such as dry cakes and cheese) to extend the shelf life and reduce the utilization of antioxidant additives (Carocho et al. 2018; Korge et al. 2020). However, aflatoxins, 3‐acetyldeoxynivalenol, deoxynivalenol, 15‐acetyldeoxynivalenol, T‐2‐toxin, ochratoxin A, zearalenone, penicylic acid, and fumonisins containing mycotoxins have been reported in 321 different chestnuts collected in China (Liang et al. 2021). Since some mycotoxins show strong carcinogenic, genotoxic, and mutagenic properties, the presence of mycotoxins adversely affects human health even at trace levels (Adenitan et al. 2021).

Zebrafish ( *Danio rerio* ), embryos and larvae are extensively used in developmental toxicology screenings (Köktürk 2022; Lin et al. 2022). Recently, zebrafish have been widely preferred to investigate the toxicities of plants in pharmacological studies (de Sá Hyacienth et al. 2020; Mhlongo et al. 2022). There are important advantages of choosing zebrafish in experimental studies (Zicarelli et al. 2022). At the same time, the zebrafish genome has almost 70% homology to the human genome and 84% of human disease‐related genes have been discovered in the zebrafish's genome (Howe et al. 2013).

Although the beneficial effects of chestnut fruits have been demonstrated, the presence of different mycotoxins in the chestnut plant should also be considered. However, the lack of studies on the evaluation and effects of chestnut outer shells remaining as waste has revealed that there is a need for scientific studies in this area. Therefore, in the current study, the secondary metabolites, antioxidant, in vivo and in vitro toxic effects of chestnut shell extract were investigated.

## 2 Materials and Methods

### 2.1 Chemicals

1,1‐Diphenyl‐2‐picryl‐hydrazyl (DPPH), butylated hydroxyanisole (BHA), neocuproine (2,9‐dimethyl‐1,10‐phenanthroline), ascorbic acid, butylated hydroxytoluene (BHT), Trolox, α‐tocopherol, Folin Ciocalteu reactive, and 5,5′‐dithio‐bis‐(2‐nitrobenzoic acid) (Ellman's Reagent) were obtained from Sigma‐Aldrich GmbH, Steinheim, Germany. Standard phenolics for LC–MS/MS were obtained from Sigma. The analysis of the plant by the LC‐ESI‐MS/MS used ultra‐grade purified methanol, water, and ammonium format obtained from Sigma Aldrich. Filters (25 mm diameter, 0.50 μm pore size, Agilent) were used for sample filtration.

### 2.2 Chestnut Material and Extractions

According to planting area, chestnuts were categorized as *Castanea americana* , *Castanea sativa* , *Castanea mollissima* , and *Castanea crenata* (Tang et al. 2007). *Castanea sativa* is well‐known in Anatolia. It is a broad‐leaved and multi‐purpose forest tree that is mostly found in the Mediterranean basin and used in the production of dried fruits, candied chestnut, chestnuts, and wood. The chestnut shells discarded in the production of chestnut candy were used in this study. The shells (100 g) were boiled in distilled water (500 mL) for 1 h. The aqueous phase was then lyophilized to dry and powdered. The final powder was stored in the refrigerator. All analyses, including LC‐ESI‐MS/MS and bioactivity assays, were conducted on reconstituted chestnut extract powder dissolved in appropriate solvents.

### 2.3 Analysis of Components in Chestnut by Using LC‐ESI‐MS/MS

For LC‐ESI‐MS/MS analysis, 100 mg of the freeze‐dried water extract of chestnut ( *Castanea mollissima* ) was accurately weighed and re‐dissolved in 20 mL of distilled methanol at room temperature (~25°C). The solution was homogenized and subsequently filtered through a 0.50 μm polytetrafluoroethylene (PTFE) syringe filter to remove particulates prior to analysis. The chromatographic separation and mass spectrometric detection were carried out using an Agilent 6460 Triple Quadrupole LC–MS system (Agilent Technologies, Santa Clara, CA, USA) equipped with an Agilent 1200 Series HPLC and an electrospray ionization (ESI) source operated in negative and positive ion modes. A Zorbax Eclipse Plus C18 column (100 × 2.1 mm, 1.8 μm particle size; Agilent Technologies) was employed for optimal compound separation. A 4 μL aliquot of the prepared sample (diluted to 2 mg/mL in LC–MS‐grade water: methanol (1:1, v/v) containing 0.1% formic acid and 5 mM ammonium formate) was injected into the system. The mobile phase consisted of Solvent A (0.1% formic acid in water with 5 mM ammonium formate) and Solvent B (methanol). A gradient elution program was applied as follows: 0–2 min: 10% B, 2–10 min: linear increase to 90% B, 10–12 min: held at 90% B, 12–13 min: returned to 10% B and 13–15 min: equilibration at 10% B. The total run time was 15 min, and the flow rate was maintained at 0.400 mL/min. The column temperature was kept constant at 30°C, and the autosampler was set to 10°C to maintain sample stability. The ESI source parameters were set as follows: Drying gas (nitrogen) flow: 10 L/min, Drying gas temperature: 300°C, Nebulizer pressure: 45 psi, Capillary voltage: 3500 V (positive mode) /−3000 V (negative mode). Multiple reaction monitoring (MRM) mode was used for detection of target phenolic compounds based on specific precursor/product ion transitions. Identification and quantification were achieved by comparing retention times and fragmentation patterns with those of analytical standards. The major phenolic compounds detected included catechin, gallic acid, taxifolin, and epicatechin. All samples were analyzed in triplicate to ensure reproducibility and accuracy (Başar et al. 2024; Yırtıcı et al. 2022).

### 2.4 Cell Culture (Caco‐2 Cells, LNCaP Cells, MDA‐MB‐231 Cells, A549 Cell and HUVEC)

LnCaP, CaCo‐2, MDA‐MB 231, A549, and HUVEC cells (European Collection of Cell Cultures, ECACC, UK) were grown in DMEM or RPMI medium supplementing 10% fetal bovine serum, antibiotics (100 U/mL penicillin, and 0.1 mg/mL streptomycin mixture) in a CO_2_ (5%) at 37°C They were subcultured for 2–3 days. The effects of water extract of chestnut were evaluated on cancer and non‐cancerous cell lines by 3‐(4,5‐dimethyltiazole‐2‐yl)‐2,5‐diphenyltetrazolium bromide (MTT) assay as detailed previously (Daikh et al. 2020). Briefly, 3000 cells/well were seeded in 96‐well plates. After a day, cells were reacted with several concentrations of chestnut extract. Extract‐treated cells were kept for a day at 37°C in a humidified CO_2_ atmosphere (5%). Then, medium was removed, and 15 μL of the MTT reagent containing fresh medium was added to each well and kept for 4 h. After incubation, medium was disposed of and 0.05 mL DMSO was transferred to each well. Then, the absorbance was recorded at 590 nm by ELISA microplate reader (Epoch, BioTek). All tests were performed three times. Viability was given as a control's percentage. Cell viability was given as described previously (Konus et al. 2020).

### 2.5 Zebrafish Embryo and Larvae Exposure

Wild‐type AB adult zebrafish ( *Danio rerio* ), which were used in this study, were obtained from the Center of Izmir Biomedicine and Genome. The study conditions and fish environments were organized and designed according to the previous study (Kokturk et al. 2022). Zebrafish larvae were selected as younger than 120 h post fertilization (hpf), after approving a permission from the ethics committee (Directive 86/609/EEC and EU Directive, 2010/63/EU). Exposure to zebrafish embryos was arranged as a semistatic test (Ensibi et al. 2014). Trial solutions were refreshed every 24 h. The trial was formed from 7 groups in which control and six different doses of chestnut (3.9, 7.8, 15.6, 31.3, 62.5, and 125 μg/mL) fruit plant extract were applied. In order to prepare different doses of chestnut fruit plant extract, a stock solution (1.0 mg/mL) was prepared with ultrapure water. Trial concentrations (3.90, 7.80, 15.60, 31.30, 62.50, and 125.00 μg/mL) from the stock solution were taken with E3 medium including KCl (0.20 mM), MgSO_4_ (0.35 mM), NaCl (5 mM), and CaCl_2_ (0.35 mM). The experiment was performed in 3 replications using 30 embryos for each group. Petri dishes were used in the experiment, and the temperature was set to 28°C. At the end of the experimental period, all zebrafish larvae used in the study were euthanized with an overdose of tricaine methanesulfonate (MS‐222) at a concentration of 300 mg/L. This method is widely recognized as humane for euthanizing zebrafish larvae. After euthanasia, the larvae were disposed of following institutional biosafety and ethical protocols.

### 2.6 Survival, Malformations, and Hatching Rates of Zebrafish Embryo and Larvae

Embryos and larvae of Zebrafish in the control and administration groups were imaged at 24 to 96 h using an SZX16 Olympus Stereomicroscope (with an SC_5_0 Olympus Camera). While the heartbeat and morphological status of embryos were examined between 24 and 96 hpf to determine mortality and malformation rates, the hatching rate was followed between 48 and 96 hpf (Köktürk et al. 2021).

### 2.7 Antioxidant Activity

#### 2.7.1 Fe^3+^ Reducing Assays

Ferric ions (Fe^3+^) reducing effects of water extract of chestnut were achieved according to previous investigations (Aytac et al. 2023; Gülçin and Alwasel 2025). For this aim, 1 mL of distilled water, and different concentrations of water extract of chestnut (10–30 μg/mL) transferred to 1.25 mL sodium phosphate buffer solution (0.2 M, pH 6.6) and 1.25 mL of K_3_[Fe(CN)_6_] (1%). After a short period (30°C), the reaction mixture was treated with 1 mL TCA (10%) and re‐incubated for half an hour in the dark. Finally, 1 mL of FeCl_3_ (0.1%) was transferred to the mixture. Then, the absorbance values of water extract of chestnut and standards were spectrophotometrically measured at 700 nm. The high absorbance of the mixture exhibits an increased reduction ability of water extract of chestnut (Guven et al. 2023).

#### 2.7.2 Cu^2+^ Reducing Assays

The cupric ions (Cu^2+^) reducing effect of water extract of chestnut was utilized in order to apply Apak et al. (2022) and give details (Bursal et al. 2020). For this aim, 0.3 mL CuCl_2_ solution (10 mM), 0.3 mL neocuproine (10 mM) and 0.3 mL CH_3_COONa solution (1.0 M) were mixed in a test tube. Then, the solution was transferred to the water extract of chestnut (10–30 μg/mL). Then, the total volumes were adjusted to 1.5 mL and kept at 25°C. Finally, the absorbances of the water extract of chestnut and standards were recorded at 450 nm (Shimadzu, UV‐1280, Kyoto, Japan).

#### 2.7.3 FRAP Reducing Ability

FRAP reducing capability of water extract of chestnut was performed according to the previous study (Durmaz et al. 2022). FRAP reagent contains acidic FeCl_3_ (10 mM) and sodium acetate solutions (0.20 mM, pH 3.6) and is prepared before use. Then, 0.5 mL of the samples, which include different concentrations of water extract of chestnut in buffer, was mixed with an equal volume of 20 mM FeCl_3_ and FRAP reagent, resulting in a 5 mL final reaction volume. Their absorbance of each reaction was calculated at a wavelength 593 nm after 30 min incubation at 37°C (Bursal and Gülçin 2011). BHT, ascorbic acid, Trolox, BHA, and α‐Tocopherol were utilized as standard molecules.

#### 2.7.4 DPPH∙ Scavenging Activities

DPPH∙ scavenging capacity of water extract of chestnut was evaluated according to the Blois technique (1958) as previously detailed (Zengin et al. 2024). Briefly, 0.5 mL of water extract of chestnut in ethanol (10–30 μg/mL) and 0.5 mL of DPPH∙ solution (0.3 mM) in ethanol were transferred to test tubes, then completed with 1 mL ethyl alcohol. The solution was incubated at 37°C for 40 min. Then, DPPH∙ removing effects of water extract of chestnut were recorded at 517 nm. The high absorbance of the reaction demonstrates increased DPPH∙ scavenging ability (Gulçin and Alwasel 2023).

#### 2.7.5 ABTS ^•+^ Scavenging Activities

ABTS^•+^ scavenging ability of water extract of chestnut was recorded according to Re et al. (1999), as given in detail previously (Guven et al. 2024). The ABTS^•+^ was generated by the reaction of 2.45 mM potassium persulfate (K_2_S_2_O_8_) with 2 mM aqueous ABTS. It was kept in the dark at 25°C for 6 h. Then, the created ABTS^•+^ solution was diluted with phosphate buffer (pH 7.4, 0.1 M) to acquire an absorbance of 0.750 ± 0.025 at 734 nm. Finally, 1000 μL of ABTS^•+^ solution was transferred to 3 mL of different concentrations of water extract of chestnut in ethanol (10–30 μg/mL). After 30 min, their absorbance was calculated at 734 nm.

### 2.8 Cholinergic Enzymes (AChE/BChE) Inhibition Assays

AChE/BChE inhibition capability of water extract of chestnut was performed according to Ellman et al.'s (1961) assay as described previously (Mahmudov et al. 2022; Gulçin, Gören et al. 2020). The AChE enzyme used in our study was obtained from electric eel ( *Electrophorus electricus* ) and the BChE enzyme was obtained from horse serum (Gul et al. 2016; Taslimi et al. 2019). Acetylthiocholine iodide (AChI) and butyrylcholine iodide (BChI) substrates were used for both reactions. If the experimental part is briefly explained, 150 μL of Tris/HCl buffer (pH 8.0, 1.0 M) and different water extract of chestnut concentrations were added to 60 μL of each cholinesterase solution (5.30 × 10^−3^ EU) and kept at 20°C for 20 min. Then, 100 μL of DTNB (0.5 mM) and 100 μL of AChI or BChI were added to the reaction mixture. The reactions were started and AChE or BChE activities were recorded at 412 nm. Also, one AChE or BChE enzyme unit is defined as the enzyme quantity that hydrolyzes AChI (1.0 mol) or BChI (1.0 mol) to choline and acetate or butyrate per minute at pH 8.0 and 37°C (Kuzu et al. 2019).

### 2.9 α‐Glycosidase Inhibition Assay

The inhibitory effects of water extract of chestnut on α‐glycosidase (from *Saccharomyces cerevisiae* ) were determined according to the method of Tao et al. (2013) as described previously (Gulçin 2020; Taslimi et al. 2021). The p‐nitrophenyl‐D‐glucopyranoside (p‐NPG) was used as a nonphysiological α‐glucosidase substrate (Tohma et al. 2016). If the experimental section is briefly explained, 100 μL phosphate buffer (pH 7.4) was added to 10 μL sample and 40 μL α‐glycosidase in phosphate buffer (0.15 U/mL, pH 7.4). Then, 100 μL of p‐NPG in phosphate buffer (pH 7.4, 5 mM) was added and the solution was kept at 37°C for 20 min. Then, the samples' absorbance was recorded at 405 nm. The amount of α‐glycosidase, which catalyzes p‐NPG (1.0 mol) per minute (pH 7.4) is accepted as one α‐glycosidase unit (Oztaskin et al. 2019; Gulçin, Trofimov et al. 2020).

### 2.10 Determination of IC_50_ Values

From plots of activity (%) versus water extract of chestnut, the half maximal inhibition concentration (IC_50_) was determined (Gulçin and Alwasel 2022).

### 2.11 Statistical Analysis

SPSS (version 25.0 and GraphPad Prism 8 statistical) software was used to determine the data analysis. In addition, all data were given as mean ± standard deviation. Tukey test was used for comparisons between experimental groups. ****p < 0.0001, ***p < 0.001, **p < 0.01, and *p < 0.05 were considered statistically significant.

## 3 Results and Discussion

### 3.1 Components Analyzed in Water Extract of Chestnut

**FIGURE 1:**
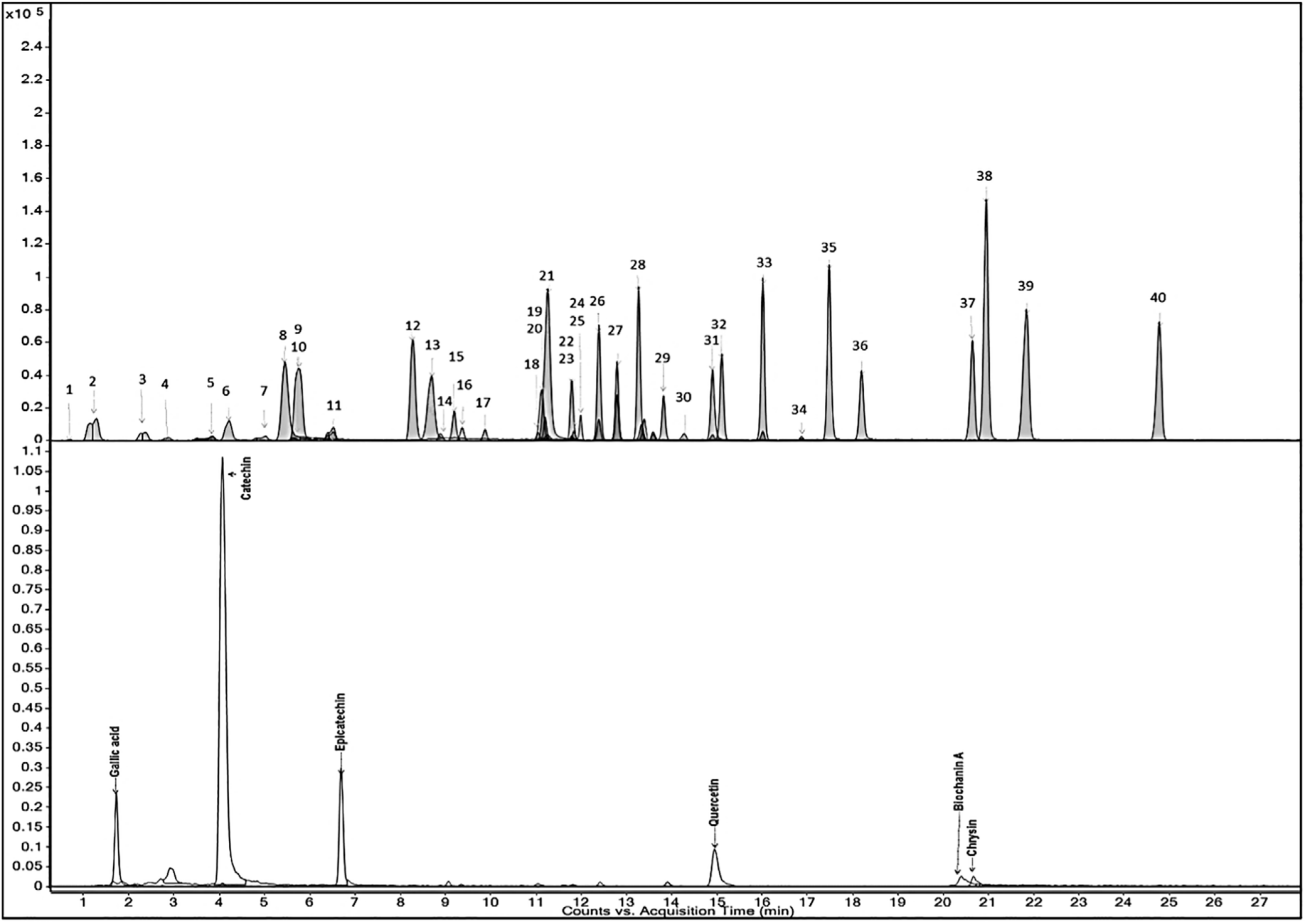
Standard compounds screened by MRM method and analysis of compounds detected in water extract of chestnut. 1‐Shikimic acid, 2‐Gallic acid, 3‐Protocatechuic acid, 4‐Gentisic acid, 5‐Catechin, 6‐4‐Hydroxybenzoic acid, 7‐Chlorogenic acid, 8‐4‐Hydroxybenzaldehyde, 9‐Vanillic acid, 10‐Caffeic acid, 11‐Epicatechin, 12‐p‐coumaric acid, 13‐Salicylic acid, 14‐Taxifolin, 15‐Polydatin, 16‐trans‐ferulic acid, 17‐Sinapic acid, 18‐Scutellarin, 19‐o‐coumaric acid, 20‐Cynarin, 21‐Protocatechuic ethyl ester, 22‐Quercetin‐3‐glucoside, 23‐Rutin, 24‐Resveratrol, 25‐Rosmarinic acid, 26‐Hesperidin, 27‐Neoesperidin, 28‐Baicalin, 29‐Kaempferol‐3‐glucoside, 30‐Fisetin, 31‐*trans*‐cinnamic acid, 32‐Quercetin, 33‐Naringenin, 34‐Hesperetin, 35‐Kaempferol, 36‐Tamarixetin, 37‐Baicalein, 38‐Biochanin A, 39‐Chrysin, 40‐Flavone, 41–6,2,4‐Trimetoxyflavone.

The compounds in the water extract of chestnut were analyzed with a new multiple reaction monitoring (MRM) method in the LC–MS/MS system. All compounds in Figure 1 were screened by this method, and gallic acid, catechin, epicatechin, taxifolin, polydatin, quercetin‐3‐glucoside, rosmarinic acid, quercetin, and chrysin compounds were found among these compounds. According to the analysis results, epicatechin (12798.886 mg/kg), gallic acid (1967.430 mg/kg), taxifolin (866.314 mg/kg), polydatin(121.014 mg/kg), epicatechin (116.010 mg/kg), and chrysin (105.968 mg/kg) in the plant extract compounds were found in high amounts, respectively (Table 1).

**TABLE 1:** Phenolic components detected in water extract of chestnut.

Codes	Name	RT	Chestnut extract (mg/kg)
1	Gallic acid	1.743	1967.430
2	Catechin	4.154	12798.886
3	Epicatechin	6.833	116.010
4	Taxifolin	9.107	866.314
5	Polydatin	9.741	121.014
6	Quercetin‐3‐glycoside	11.908	6.723
7	Rosmarinic acid	12.168	75.048
8	Quercetin	15.033	54.889
9	Chrysin	20.847	105.968

Chestnut attracts more attention in terms of its use in the field of health, as it contains important active compounds including catechin, gallic acid, and taxifolin. Similarly, studies have determined high antioxidant activity, total phenolics (131.84 mg gallic acid equivalent/100 g FW) and total flavonoid compounds (7.77 mg eq. catechin/100 g) in chestnuts (Martínez et al. 2022). It is known that these chemicals, which are detected in high amounts, have a vital role in cancer, cardiovascular, and neurodegenerative diseases (Bernatoniene and Kopustinskiene 2018). Additionally, low levels of epicatechin, polydatin, quercetin‐3‐glucoside, rosmarinic acid, quercetin, and chrysin compounds also have important bioactive effects (Li et al. 2016; Yırtıcı et al. 2022).

### 3.2 Cell Toxicity

**FIGURE 2:**
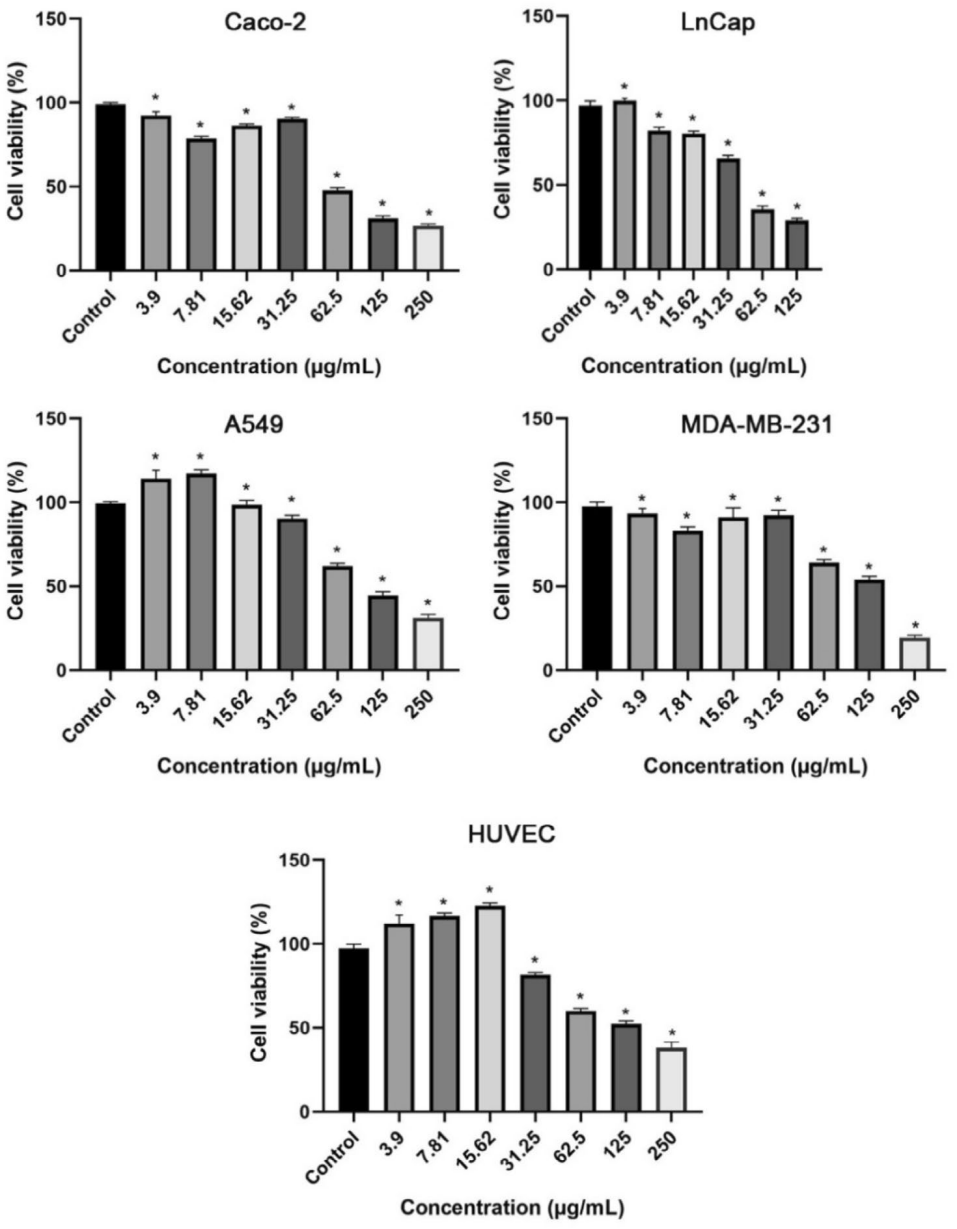
The cytotoxic effects of chestnut water extract on in MDA‐MB 231, CaCo‐2, LnCaP, A549, and HUVEC cell lines determined by MTT analysis *p < 0.05.

The cytotoxicity of chestnut extract was evaluated in LnCaP, MDA‐MB 231, CaCo‐2, A549, and HUVEC cell lines by MTT test as described in methods. The EC_50_ values of the extract were 64.1 μg/mL for LnCaP, 99.6 μg/mL for CaCo‐2, 101.4 μg/mL for MDA‐MB 231, 152 μg/mL for A549, and 152 μg/mL for HUVEC cells, respectively (Figure 2). Chestnut extract had a dose‐dependent cytotoxic effect in all tested cell lines. These results exhibited that the extract is more efficient in terms of cytotoxicity towards the prostate cancer cell line. Moreover, these results demonstrated that chestnut extract has more cytotoxic ability towards LnCaP, CaCo‐2, and MDA‐MB‐231 cell lines than that of the non‐cancerous cell (HUVEC). Further studies on that extract and its secondary metabolites showing selectivity for certain cancers and their action mechanisms might be valuable. Similar to our observation, different solvent extracts prepared using different parts of chestnut showed cytotoxic effects on different cell lines (Sapkota et al. 2010; Cacciola et al. 2020).

### 3.3 Zebrafish Developmental Toxicity (Survival Rate, Malformations Rate, and Hatching Rate)

**FIGURE 3:**
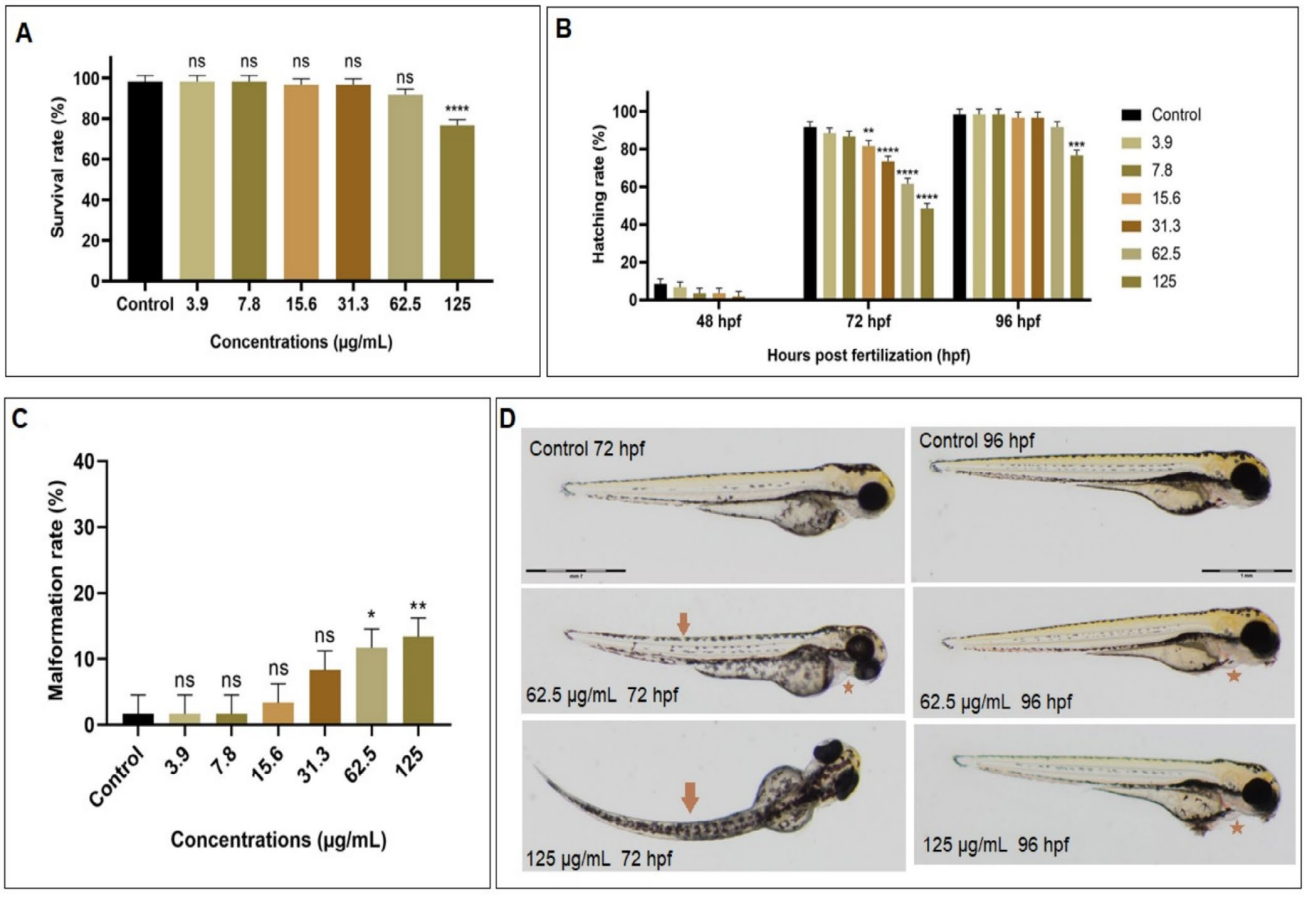
Survival rate (A) and Hatching rate (B), Malformation rate C) and microscope images of malformations (D) of zebrafish embryos and larvae are subject to different doses (3.9, 7.8, 15.6, 31.3, 62.5, and 125 μg/mL) of *Castanea sativa* Mill during 96 hpf. Data were expressed as mean ± SD (*n* = 30/group). ****p < 0.0001, ***p < 0.001, **p < 0.01, and *p < 0.05 as compared with the control. Brown star: Pericardial edema, brown arrow: Spinal curvature. Scale:1 mm.

The developmental toxicity was evaluated by exposing the fruit part of the water extract of chestnut at different doses (3.9, 7.8, 15.6, 31.3, 62.5, and 125 μg/mL) in embryos and larvae of zebrafish for 96 h after fertilization. In our study, chestnut plant application concentrations on zebrafish embryos and larvae were selected similar to cell culture application groups in order to interpret the in vivo and in vitro effects. Survival, hatching, and malformation rates were determined after the chestnut fruit was exposed to embryos and larvae for 96 h. It was exhibited that there was a meaningful difference with a 76.7% survival rate in embryos and larvae when checked against the control group at the highest application dose (125 μg/mL), and it was determined that the highest mortality rate was at this dose (Figure 3A) (****p < 0.0001). It was found that the rate of emergence of embryos from the chorion decreased at the highest dose at the 96th hour compared to the control (Figure 3B) (***p < 0.001). Larval hatching rates were found to be > 90% in all treatment groups except 125 μg/mL (Figure 2B). It has been found that the chestnut plant causes morphological changes such as pericardial edema and spinal curvature in embryos and larvae of zebrafish (Figure 3D). Significant differences in disability rates were found at the 62.5 and 125 μg/mL doses compared with the control, and the malformation rates were 11.7% and 13.3%, respectively (Figure 3C) (**p < 0.01, and *p < 0.05).

A study on the human keratinocyte cell line (HaCaT) exhibited that the protective effect of water extract of chestnut against DNA damage reveals the importance of antioxidant capacity (Almeida et al. 2015). It has been determined that *Castanea sativa* Mill type chestnuts have beneficial effects on intestinal epithelial cells by increasing their antioxidant potential and not affecting cellular metabolism (Brus et al. 2018). In a prior study, it was found that low concentrations of *Castanea sativa* Mill plant did not cause toxicity in the development of HUVEC cell culture and zebrafish embryo‐larval model. In this case, we can say that the chestnut plant does not show negative effects on cell pathways in vivo and in vitro at low application doses.

Some cytotoxicity studies with extracts from different parts of the chestnut have been mentioned above. However, in vivo toxic effects of *Castanea sativa* Mill extracts on zebrafish embryos and the probable for developmental toxicity of embryo‐larvae at early developmental periods have not yet been reported. In general, the zebrafish model is widely used for evaluation the possible toxic effects of plant extracts (Modarresi Chahardehi et al. 2020; Zainol Abidin et al. 2020). In this study, it was shown that there was an increasing mortality rate in the highest dose application group in embryos and larvae and that the success of the larvae exiting the chorion decreased at high concentrations depending on the exposure time of the chestnut plant. Especially in groups with low chorion exit success, embryos had different morphological disorders (spinal curvature) and these malformations affected embryo movement and the result was delayed larval hatching. Delays and disruptions in morphological development, such as spine and musculature of zebrafish embryos, prevent hatching (Wu et al. 2022; Jablonski et al. 2022). Embryos need to make spontaneous movements in order to break out of their chorionic membranes (Sano et al. 2008). In this study, it was exhibited that the larval hatching rate decreased due to the decrease in the number of spontaneous movements in zebrafish embryos exposed to high concentrations of *Castanea sativa* Mill extract. Similarly, it has been found that spontaneous movements in zebrafish embryos are affected by the effect of some plants (Abd Rashid et al. 2022).

Chestnut is an important food with high antioxidant capacity and high bioactive compound content. Chestnut attracts more attention in terms of its use in the field of health, as it contains important active compounds such as catechin and gallic acid (Mustafa et al. 2021). The biological effects of such active compounds, such as amelioration of cancer (Patra et al. 2021), cardiovascular (Sanches‐Silva et al. 2020) and neurodegenerative diseases (Islam et al. 2022) have been proven. Previous studies have determined high antioxidant activity, total phenolic compounds (131.84 mg gallic acid equivalent/100 g FW) and total flavonoid compounds (7.77 mg eq. catechin/100 g) in chestnuts (Martínez et al. 2022). Similarly, high amounts of catechin and gallic acid were detected in Castanea extract in our study. In addition, important bioactive compounds were analyzed.

In our study, chestnut extract had a dose‐dependent cytotoxic effect in all tested cell lines. These results demonstrated that the extract is more efficient in terms of cytotoxicity against the prostate cancer cell line. Moreover, these results demonstrated that chestnut extract has a more cytotoxic effect towards LnCaP, Caco‐2, and MDA‐MB‐231 celllines than non‐cancerous cells (HUVEC) but it was found that Castanea extract did not cause toxicity at similar low concentrations on the HUVEC cell line and in zebrafish embryo‐larval development.

There are studies showing that the chestnut plant has a potentially lethal effect on cancer cells, so it can be used in complementary medicine (Cacciola et al. 2020; Nascimento‐Gonçalves et al. 2021). In our study, it was defined that some low concentrations of *Castanea sativa* Mill extract had a lethal effect on the Caco‐2 cell line, and it was determined that similar doses did not have a toxic effect on zebrafish embryo‐larvae. This shows that low doses do not have negative effects on in vivo living pathways, and the use of *Castanea sativa* Mill in treating colon cancer may be promising. The effect of *Castanea sativa* Mill on colon cancer can be explained by the inhibition effect of antioxidant polyphenols, including phenolic acids, flavonoids, lignans, proanthocyanidins, and stilbenes produced in plants on the NF‐κB pathways of intestinal inflammation formed by Caco‐2 cell lines (Romier‐Crouzet et al. 2009; Peng, Guo et al. 2022). It is also known that natural polyphenols in plants have lethal effects on lung (Wang et al. 2020), breast (Selvakumar et al. 2020) and skin (Sajadimajd et al. 2020) cancers. In our study, it was found that *Castanea sativa* Mill extract has a lethal effect on lung (A549 cells) and breast cancer (MDA‐MB‐231 cells) cell lines.

### 3.4 Antioxidant Activity

Antioxidants can eliminate the undesirable negative effects that may be caused by lipid oxidation during the production and processing of foods. In this context, the antioxidants are defined as natural or synthetic substances that can significantly delay or entirely prevent the oxidation of substrates even at low use concentrations (Liang et al. 2021; Ozden et al. 2023). For this purpose, many different antioxidants are used to prevent spoilage in foods (Othón‐Díaz et al. 2023). Of these, synthetic antioxidants are generally used today due to their high purity, low cost, and very effectiveness even when used at low concentrations. However, recently, the use of these synthetic antioxidants has been seriously limited due to their undesirable negative effects (Tohma et al. 2016; Durmaz et al. 2023).

**FIGURE 4:**
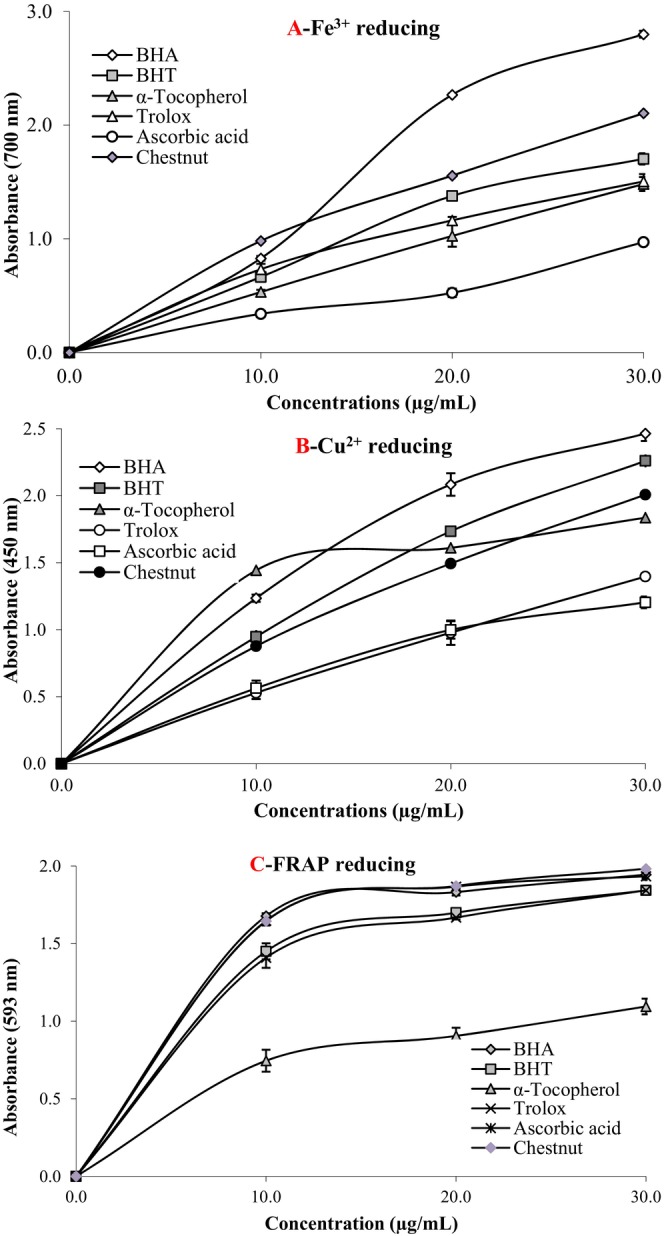
Reducing power of 10–30 μg/mL of water extract of chestnuts by ferric ions (Fe^3+^) (A), cupric ions (Cu^2+^) reducing (B), and FRAP reducing (C) capacities.

There are many different bioanalytical methods to evaluate the antioxidant effects of plant products with rich phenolic contents. Therefore, it is of great importance to select the most appropriate antioxidant analysis (Gulçin et al. 2004; Gulçin 2020). In our study, the antioxidant ability of the water extract of chestnut was investigated using five different and distinct methods with a realistic approach in terms of different antioxidant properties. Also, in this study, DPPH radicals and ABTS radicals scavenging and reducing properties, which are common and effective bioanalytical methods, were effectively used for the evaluation of the antioxidant effect of the water extract of chestnut. The reduction potentials of the water extract of chestnut were explained using three different methods, namely Fe^3+^, Cu^2+^, and Fe^3+^‐TPTZ reducing properties (Figure 4 and Table 2). In addition, DPPH∙ and ABTS∙^+^ removing methods, which are effective and common methods, were also used to reveal the radical removing effect of the chestnut's water extract. It is known that antioxidants contribute to the prevention of many diseases by clearing or neutralizing oxidative stress and reactive oxygen species accumulated in tissues during metabolic activities (Gulçin and Alwasel 2023). In our study, the antioxidant features of the water extract of chestnut were estimated through their ability to clear free radicals and reduce metals.

**TABLE 2:** Determination of reducing ability of similar concentration (30 μg/mL) of water extract of chestnuts by FRAP reducing, ferric (Fe^3+^), and cupric (Cu^2+^) ions reducing capacities.

Antioxidants	Fe^3+^‐Fe^2+^reducing		Cu^2+^‐Cu^+^ reducing		Fe^3+^‐TPTZ reducing	
	*λ*_700_	*r* ^2^	*λ*_450_	*r* ^2^	*λ*_593_	*r* ^2^
BHA	2.799 ± 0.030	0.9769	2.464 ± 0.055	0.9999	1.944 ± 0.032	0.9763
BHT	1.703 ± 0.049	0.9945	2.260 ± 0.047	0.9845	1.842 ± 0.007	0.9721
α‐Tocopherol	1.482 ± 0.061	0.9988	1.836 ± 0.020	0.9570	1.094 ± 0.050	0.9725
Trolox	1.505 ± 0.064	0.9982	1.397 ± 0.017	0.9971	1.842 ± 0.009	0.9730
Ascorbic acid	0.971 ± 0.016	0.9823	1.203 ± 0.043	0.9994	1.933 ± 0.011	0.9678
Chestnut extract	2.104 ± 0.046	0.9970	2.007 ± 0.041	0.9995	1.982 ± 0.011	0.9671

It is known that the leaves, inner bark, flowers, and nuts of chestnut varieties are very important for the human health because they contain abundant phenolic compounds and have strong antioxidant activity (Tuyen et al. 2017). The reducing capacity of the crude water extract of chestnut burs is summarized in Figure 3. The absorbance of the reaction medium was highly correlated with the concentration of water extract of chestnut (*r* ^2^: 0.9996), and the higher slope of the line indicates higher reducing effect of water extract of chestnut samples. As seen in Table 2 and Figure 4A, water extract of chestnut revealed potent Fe^3+^ reducing effect and these differences were statistically found as very important (*p* < 0.01). The reducing capacity of water extract of chestnut, BHT, α‐tocopherol, and ascorbic acid augmented frequently when the concentration of sample was increased. Fe^3+^ reducing effect of water extract of chestnut and standards exposed the following order: BHA (*λ*_700_: 2.799 ± 0.030, *r* ^2^: 0.9769) > water extract of chestnut (*λ*_700_: 2.104 ± 0.046, *r* ^2^: 0.9970) > BHT (*λ*_700_: 1.703 ± 0.049, *r* ^2^: 0.9945) > Trolox (*λ*_700_: 1.505 ± 0.064, *r* ^2^: 0.9982) > α‐Tocopherol (*λ*_700_: 1.482 ± 0.061, *r* ^2^: 0.9988) > Ascorbic acid (*λ*_700_: 0.971 ± 0.016, *r* ^2^: 0.9823) at 20 μg/mL. The results showed that water extract of chestnut had marked and powerful Fe^3+^ reducing effects. Considering the studies on this subject, 50–250 μg/mL concentrations of Chinese chestnut ( *Castanea mollissima* ) indicated higher reducing power of the samples with absorbance between 0.250 and 0.850 at 700 nm (Zhao et al. 2011).

The Cu^2+^‐reducing capacity of the water extract of chestnuts was evaluated and compared to positive controls like BHA, ascorbic acid, BHT, and Trolox. The Cu^2+^ reducing effects of the water extract of chestnuts are summarized in Table 2 and given in Figure 4B. While investigating the Cu^3+^ reduction capacities of the samples, a strong correlation was observed between the Cu^2+^ reducing ability and different water extract of chestnuts concentrations. In addition to this correlation, at 45 μg/mL, effective reducing ability was demonstrated by the water extract of chestnuts. Also, Cu^2+^ reducing ability of the water extract of chestnuts and the standards were calculated to be BHA (2.464 ± 0.055, *r* ^2^: 0.9999) > BHT (2.260 ± 0.047, *r* ^2^: 0.9845) > water extract of chestnuts (2.007 ± 0.041, *r* ^2^: 0.9995) > α‐tocopherol (1.836 ± 0.020, *r* ^2^: 0.9570) > Trolox (1.397 ± 0.017, *r* ^2^: 0.9971) > ascorbic acid (1.203 ± 0.043, *r* ^2^: 0.9994). It was observed that the water extract of chestnuts reduced cupric ions more effectively than α‐tocopherol, Trolox, and ascorbic acid, but less than BHA and BHT, which were used in the study. As explained in the previous method, samples with absorbance values higher than 1.5 in spectrophotometric measurements were diluted to obtain accurate measurements in accordance with the Lambert–Beer law. In addition, the results were multiplied by the dilution factor. In this test, the higher absorbance values reflect the greater the reducing ability of the test samples.

In terms of antioxidant activity, the reducing effect of the water extract of chestnut is considered an important antioxidant indicator. Reducing ability reflects the capability of an extract to break the free radical chain through hydrogen donation. The FRAP method measured the antioxidant abilities to reduce the ferric form of Fe^3+^‐TPTZ to its ferrous form of Fe^2+^‐TPTZ due to their reductive potential. The change pattern of the FRAP value was similar to that of the other reducing ability assays. The water extract of chestnut demonstrated effective reducing ability in a dose‐dependent manner. As given in Table 2, Figure 4C, the Fe^3+^‐ferricyanide complex reducing ability decreased with an increasing concentration of chestnut extract, which was similar to the standard antioxidants. The reducing powers of the same concentration extract and the standards were observed to decrease as follows: chestnut extract (1.982 ± 0.011, *r* ^2^: 0.9671) > BHA (1.944 ± 0.032, *r* ^2^: 0.9763) > ascorbic acid (1.933 ± 0.011, *r* ^2^: 0.9678) > BHT (1.842 ± 0.007, *r* ^2^: 0.9721) ≈ Trolox (1.842 ± 0.009, *r* ^2^: 0.9730) > α‐tocopherol (1.094 ± 0.050, *r* ^2^: 0.9725).

**FIGURE 5:**
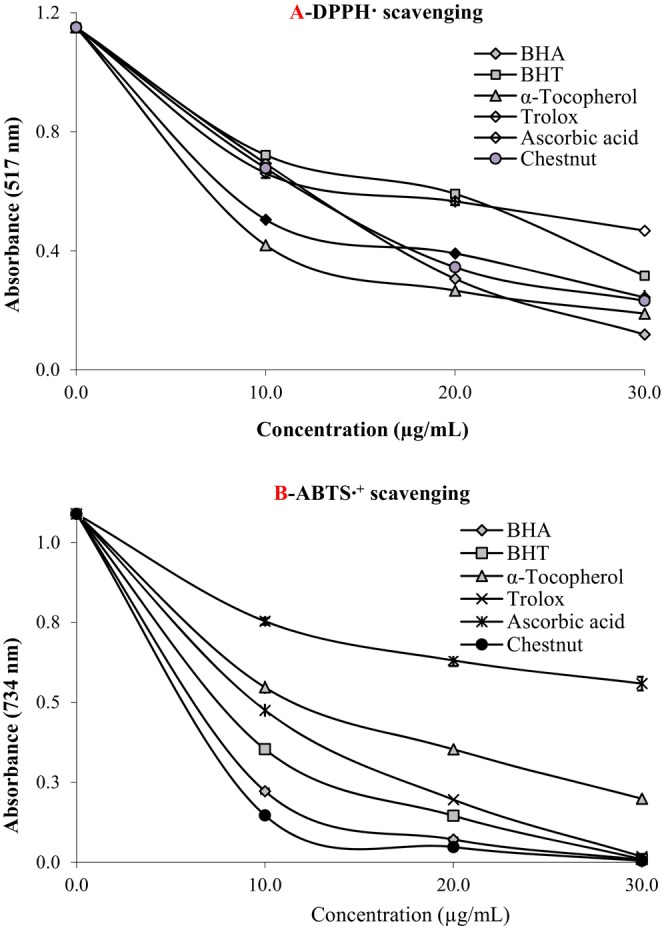
Radical scavenging activities of 10–30 μg/mL of water extract of chestnuts on (A) DPPH and (B) ABTS^•+^ scavenging activities.

The radical scavenging effects of chestnut water extract were evaluated using the ABTS radical scavenging test according to Zhou et al. (2011) and the DPPH radical scavenging method established by Blois (1958). For this purpose, experimental studies were carried out using chestnut extract in different concentrations. In radical scavenging ability, our findings demonstrated that chestnut extract had a lower IC_50_ value (12.60 μg/mL, *r* ^2^:0.9996). The lower the IC_50_ value reflects the higher the reducing power and antioxidant ability. The data obtained show that chestnut extract was more effective than BHT (IC_50_: 12.90 μg/mL, *r* ^2^:0.9729), Trolox (IC_50_: 12.72 μg/mL, *r* ^2^:0.9666) and Ascorbic acid (IC_50_: 21.00 μg/mL, *r* ^2^:0.9712) and its DPPH radical scavenging ability was close to BHA (IC_50_: 9.76 μg/mL, *r* ^2^:0.9986) and α‐Tocopherol (IC_50_: 10.34 μg/mL, *r* ^2^:0.9782) (Table 3, Figure 5A). DPPH radicals are quenched by antioxidants that can provide hydrogen and are the most used method for determination of antioxidant activity. Thus, the chestnut extract had efficient scavenging ability against DPPH free radicals. In another study, Campo et al. (2016) indicated that sweet chestnut (*Castanea saliva*) wood extracts had an IC_50_ value of 4.55 ± 0.05 mg/mL, which is higher than the IC_50_ values found in this study. Free radicals are known as hazardous agents that can damage tissues via oxidative stress. This stress plays a vital role in the development of many diseases (Zhang et al. 2022). In another study, it is known that Chinese chestnut ( *Castanea mollissima* ) is a product of economic value and is widely grown in Europe, North America, and Asia, effectively removes DPPH radicals. In this study, the water extract of chestnut burs demonstrated considerable DPPH radical scavenging effects with an IC_50_ of 50.9 μg/mL (Zhao et al. 2011). In a recent study, it was shown that *Castanea sativa* extracts fractionated by Sephadex LH‐20 chromatography had IC_50_ values between 2.8 and 20.1 μg/mL for DPPH removing activity (Cardullo et al. 2018). Other studies of this bark, inner skin, flower, kernel, and leaf extracts of *Castanea crenata* exhibited DPPH radical scavenging potency in a range of 23.81 to 48.98 μg/mL (Tuyen et al. 2017).

**TABLE 3:** Determination of IC_50_ of water extract of chestnuts and standards for DPPH· and ABTS^•+^ scavenging activities.

Antioxidants	DPPH· scavenging		ABTS^•+^ scavenging	
	IC_50_	*r* ^2^	IC_50_	*r* ^2^
BHA	9.76	0.9986	4.39	0.9984
BHT	16.90	0.9729	4.98	0.9926
α‐Tocopherol	10.34	0.9782	12.16	0.9894
Trolox	12.72	0.9666	5.82	0.9959
Ascorbic acid	21.00	0.9712	27.72	0.9919
Chestnut extract	12.60	0.9996	3.89	0.9980

Alzheimer's disease (AD) is a globally known neurodegenerative disease that affects approximately 40 million people worldwide, causing serious concerns. AD forms due to losing neurons and synapses in different parts of the central nervous system (Taslimi et al. 2017; Genc Bilgicli et al. 2019). Inhibition of cholinesterase enzymes has a very important target in the field of potential therapeutics for the treatment of AD (Ozbey et al. 2016; Tohma et al. 2019). In particular, cholinergic enzymes such as AChE and BChE play a very important role in terminating cholinergic neurotransmission by assisting the hydrolysis of acetylcholine (ACh). ACh levels decrease with advancing age in AD patients. Accordingly, the use of AChE and BChE inhibitors has been shown to effectively alleviate AD symptoms (Turan et al. 2016; Alves et al. 2022). The usage of AChE and BChE inhibitors is the most effective strategy for AD treatment by lowering ACh concentration. Both cholinesterase inhibitors including tacrine, donepezil, galantamine, rivastigmine, and memantine only provide a temporary improvement of AD symptoms (Sujayev et al. 2016; Topal et al. 2017). Especially, tacrine has some significant and undesired side effects including nausea, diarrhea, vomiting, and headache (Kozurkova et al. 2011). So, scientific research and discoveries for new and natural cholinesterase inhibitors for the AD treatment continue intensively (Scozzafava et al. 2015; Demir et al. 2019). Also, in the present study, the water extract of chestnuts was tested at different concentrations in order to determine its inhibitory effects on both enzymes (Table 4). Evaluation of the inhibitory effect of chestnut extract on both cholinergic enzymes has provided valuable information about their neuroprotective potential. IC_50_ values were also recorded to compare the inhibitory effects of all applications towards both enzymes. The results obtained from the current study exhibited that the water extract of chestnut demonstrated AChE (IC_50_:0.307 μg/mL, *r* ^2^: 0.9770) and BChE (IC_50_:0.152 μg/mL, *r* ^2^: 0.9842) inhibition potency when compared with Tacrine (IC_50_:0.084 μg/mL, *r* ^2^: 0.9881) as commercial inhibitors (Table 4, Figure 5B).

**TABLE 4:** IC_50_ values (μg/mL) of water extract of chestnuts toward AChE, BChE, and α‐glycosidase.

Enzymes	Chestnut water extract		Standards	
	IC_50_ (μg/mL)	*r* ^2^	IC_50_ (μg/mL)	*r* ^2^
AChE	0.307	0.9770	0.084	0.9881
BChE	0.648	0.9902	0.152	0.9842
α‐Glycosidase	0.473	0.9797	—	—

Diabetes mellitus (DM) is a metabolic and common disease characterized by hyperglycemia resulting from insulin secretion, insulin action, or both conditions (Yamali et al. 2018). DM is a disease that affects many organs, especially the eyes and kidneys, and causes major complications (Mirazi and Hosseini 2020). Today, the most‐known approach to treating diabetes is to reduce postprandial hyperglycemia by inhibiting the main digestive enzyme, α‐glycosidase (Oboh et al. 2015; Taslimi and Gulçin 2017), to delay or prevent glucose absorption from the small intestines. Therefore, in this study, the inhibition effect of the water extract of chestnut towards α‐glycosidase enzyme was determined to evaluate its antidiabetic potential. Also, the inhibition effect of the water extract of chestnut was compared with the effect of acarbose, which is also used as an antidiabetic agent (Table 4). As shown in Table 4, the water extract of chestnut exhibited significant inhibition effects against α‐glycosidase activity with an IC_50_ of 0.473 μg/mL (*r* ^2^: 0.9797). However, it was recorded that acarbose, which is a pure substance, demonstrated an IC_50_ of 22,800 μM (Taslimi et al. 2017). In a prior study, it was reported that *Castanea sativa* extracts fractionated by Sephadex LH‐20 chromatography had IC_50_ values between 8.3 and 74.1 μg/mL against yeast α‐glucosidase (Cardullo et al. 2018).

## 4 Conclusion

Water extract of chestnut ( *Castanea mollissima* Blume) used in the present study did not cause developmental toxicity and teratogenicity in zebrafish embryos. It was exhibited that there was a meaningful difference with a 76.7% survival rate in embryos and larvae when checked against the control group at the highest application dose (125 μg/mL), and it was determined that the highest mortality rate was at this dose (*p* < 0.0001). The larval hatching rates were found to be > 90% in all treatment groups except 125 μg/mL. These results show that the chestnut plant causes morphological changes such as pericardial edema and spinal curvature in zebrafish embryos and larvae. The study also aimed to identify potentially active phenolic compounds existing in the water extract of chestnut. LC‐HR/MS analysis performed unveiled phenolics such as catechin (12798.886 mg/kg extract), gallic acid (1967.430 mg/kg extract), taxifolin (866.314 mg/kg extract), and epicatechin (116.010 mg/kg extract) in the water extract of chestnut. On the other hand, the antioxidant ability of plants and fruits is frequently used and accepted as a criterion for bioactive compounds. Comparing water extract of chestnut's antioxidant ability to that of BHA, Ascorbic acid, BHT, Trolox, and α‐tocopherol was utilized in this study. The chestnut's fruit surpasses many commonly eaten fruits in terms of phenolic acid content. The antioxidant features of chestnut were evaluated using several bioanalytical assays, and effective results were determined. For example, the chestnut's water extract effectively exhibited DPPH· scavenging (IC_50_: 12.60 μg/mL) and ABTS radical scavenging (IC_50_: 3.89 μg/mL). Also, Also, its inhibitory effects chestnut's water extract on some key and metabolic enzymes, including AChE (IC_50_: 0.084 μg/mL), BChE (IC_50_: 0.152 μg/mL), and α‐glycosidase (IC_50_: 0.473 μg/mL) related to diabetes and AD are very important. These findings strongly show that the chestnut's water extract can serve as a valuable source of antioxidant molecules that are crucial for effects in biological functions. The existence of polyphenolic compounds in the water extract of chestnut plays quite an important role for their antioxidant, anti‐Alzheimer's disease, and antidiabetic abilities.

## Author Contributions

**İbrahim Demirtas:** conceptualization, investigation, supervision. **Mehmet Nuri Atalar:** investigation. **Zeynebe Bingol:** investigation. **Mine Köktürk:** investigation, writing – original draft. **Gunes Ozhan:** investigation. **Amine Hafis Abdelsalam:** investigation. **Sevki Arslan:** investigation. **İlhami Gülçin:** writing – original draft, conceptualization, supervision.

## Conflicts of Interest

The authors declare no conflicts of interest.

## Data Availability

The authors have nothing to report.
